# Video-based examination of patients with shoulder pain: a scoping review

**DOI:** 10.1186/s12891-026-10243-y

**Published:** 2026-07-20

**Authors:** Pernilla Pålsson, Viktor Ljung, Susanne Bernhardsson, Elvira Lange

**Affiliations:** 1 Unit of Physiotherapy, Department of Health and Rehabilitation, Institute of Neuroscience and Physiology, Sahlgrenska Academy, University of Gothenburg, Gothenburg, Sweden; 2 Research, Education, Development and Innovation, Primary Health Care, Region Västra Götaland, Gothenburg, Sweden; 3 Department of General Practice / Family Medicine, School of Public Health and Community Medicine, Institute of Medicine, Sahlgrenska Academy, University of Gothenburg, Gothenburg, Sweden

**Keywords:** Video-based, Virtual, Shoulder, Examination, Assessment

## Abstract

**Background:**

Virtual healthcare has developed rapidly and become an important part of medical care. Shoulder complaints are a common reason for seeking care and require thorough examination. Studies indicate that musculoskeletal examinations can be performed via video with good validity, but the evidence is limited. There is a need to explore, identify and map current research on the topic. The aim of this scoping review was to map and compile the existing literature on real-time video-based examination of the shoulder, to identify knowledge gaps in this area, and to describe procedures used in shoulder examinations.

**Methods:**

A literature search was conducted in November 2024 on PubMed, CINAHL and Scopus, and updated in June 2026. Grey literature was excluded. Selection of literature was performed independently by two authors. Conduct and reporting of the review followed established guidance for scoping reviews.

**Results:**

Twenty articles were included in the review. Seven were designed as guidelines, 12 were validity or reliability studies, and one article was a case report. A result was compiled that describes the different parts of a video-based examination of the shoulder. The result also includes a description of investigated validity and reliability for individual examination components, and overall clinical assessment and diagnosis.

**Conclusions:**

This scoping review identified multiple studies outlining examination components and practical procedures for video-based assessment of the shoulder, suggesting the usefulness of this mode. Nevertheless, more research is necessary to establish a robust evidence base. Further research is needed to determine best practices and the appropriate role of video-based shoulder examination in musculoskeletal care. .

**Clinical trial registration:**

Not applicable. This study is not a clinical trial.

**Clinical trial number:**

Not applicable. The scoping review was preregistered on 8 Nov 2024 on Open Science Framework Registry (Identifier: 10.17605/OSF.IO/B4C8S).

**Supplementary Information:**

The online version contains supplementary material available at 10.1186/s12891-026-10243-y.

## Background

Virtual healthcare has undergone rapid development in recent years, particularly during the COVID-19 pandemic, when a substantial increase in the use of virtual health services was observed globally [1]. By 2020, approximately half of the world’s population had access to the internet [2]. With this growing accessibility, the conditions for expanded use of virtual healthcare have also improved. Virtual healthcare refers to health services provided through virtual remote communication, where the patient and healthcare provider are physically separated. Communication may occur in real-time or asynchronously and includes tools such as telephone, video, chat, email, text messages, image messaging, and mobile applications [3]. Other terms used in this field include telehealth, telemedicine, telerehabilitation, and digital healthcare [4]. Virtual healthcare may offer several advantages, such as reduced waiting times and the removal of barriers like high costs associated with long-term treatments and long travel distances to healthcare facilities [5].

Shoulder-related disorders are the third most common musculoskeletal complaint in primary care, after back and neck disorders [6]. These conditions may involve orthopaedic injuries such as fractures and tendon or muscle ruptures, but more commonly relate to pain and limited mobility caused by conditions such as impingement syndrome or adhesive capsulitis [7]. Shoulder pain is prevalent amongst adults worldwide. Reported prevalence varies significantly depending on the time frame assessed, ranging from 11 to 55% for 12-month prevalence, and 2–34% for seven-day prevalence [8]. To determine the nature and cause of shoulder complaints and identify the affected structures, a musculoskeletal examination should be conducted. This examination should be person-centered, based on the individual’s needs and context, and include assessment of mobility, strength, posture, and, when appropriate, neurological function [9].

A musculoskeletal examination can be conducted virtually, and some studies indicate that virtual consultations may be a viable alternative to face-to-face visits [10]. Methods also exist for evaluating range of motion (ROM), pain, and certain clinical tests that demonstrate validity and reliability when performed via video consultation [10]. However, the body of evidence in this area remains limited, making it difficult to establish under what circumstances and for which conditions video-based assessments are effective. Previous reviews have addressed related areas: Zischke et al. [11] synthesized evidence on the clinimetric properties of physiotherapy assessments delivered virtually across diverse conditions, while Maltby et al. [12] mapped the broader use of telephone and video consultations in upper limb disorders. Although these reviews highlight the potential of virtual consultations, neither provides a detailed account of how video-based assessments of the shoulder are conducted in practice, leaving an important gap that the present review aimed to address. Moreover, previous reviews are based on literature searches conducted in 2020–2021, which, given the rapid development of virtual care, highlights the need for an up-to-date review of available literature. There is a paucity of studies that have specifically examined validity or agreement between video-based and in-person evaluations of the shoulder, precluding any robust conclusions [12, 13].

Since shoulder-related problems are amongst the most common complaints, more knowledge is needed on how clinical assessments of the shoulder can be practically conducted in a virtual context. Therefore, the aim of this scoping review was to map and compile the current literature on real-time, video-based musculoskeletal assessment of the shoulder and to identify current knowledge gaps. Additionally, the review aimed to describe clinical procedures used in video-based examination of the shoulder in relation to the various included components.

## Methods

The project followed the methodology for conducting a scoping review as described by Arksey & O’Malley [14] and the subsequent methodological enhancements proposed by Levac et al. [15]. The work adhered to the five stages outlined below to ensure a systematic approach and to facilitate the project process.

1. Identification of the research question
2. Identification of relevant studies
3. Study selection
4. Charting of the data
5. Collating, summarising and reporting the results

The review is reported according to the Preferred Reporting Items for Systematic reviews and Meta-Analyses extension for Scoping Reviews (PRISMA-ScR) checklist (Additional file 1) [16]. The review was preregistered on Open Science Framework, 10.17605/OSF.IO/B4C8S.

### Identification of the research question

As a first step, the research question was formulated in accordance with the PCC framework (Population, Concept, Context) [17]. The “Population” includes shoulder-related problems regardless of diagnosis (e.g., pain, instability, orthopaedic injuries). The “Concept” is limited to musculoskeletal examination (including components such as ROM, pain, muscle strength, and neurological assessment), and the “Context” refers to assessments conducted virtually with real-time audio and video communication.

### Identification of relevant studies

To locate relevant studies, literature searches were conducted on 11 November, 2024, in the databases PubMed, CINAHL, and Scopus. The search was updated on 30 June 2026, using the same search strategy (Additional file 2). Our review protocol also specified that we would search also the PEDro database and the DIVA portal. However, PEDro was not used in the final search because preliminary searches found no additional relevant studies beyond those indexed in PubMed or Scopus, and its focus on randomized trials and clinical guidelines was not aligned with the scope of our review. DIVA was not used because it primarily indexes non-peer-reviewed materials. The search strategy was based on the terms *Video-based*, Exam* and Shoulder*, along with multiple synonyms. A broad approach was used to reduce the risk of missing relevant studies, while limiting the search to scientific databases to avoid studies of questionable quality.

### Study selection

To facilitate the selection process, the web-based platform Rayyan was used [18]. Search hits were exported to Rayyan, and duplicates were removed using the platform’s built-in function. Two authors independently screened the titles and abstracts of the remaining articles. The inclusion or exclusion of articles was then discussed jointly, based on predefined inclusion and exclusion criteria, and decisions were made collaboratively. Full-text screening was conducted independently by two authors, followed by discussion to reach consensus on final inclusion. In some cases, disagreements were resolved with input from a third author.

Inclusion criteria:

- Literature describing musculoskeletal video-based examination of the shoulder, where some part of the examination is conducted in real-time.
- Articles published in peer-reviewed scientific journals, from inception to 11 November 2024. Time period for the updated search was 11 November 2024 to 30 June 2026.
- Articles written in English or Swedish.
- Literature accessible through the University of Gothenburg Library.

Exclusion criteria:

- Animal studies.
- Studies requiring patients to be in a specific setting or to have access to equipment beyond a phone, computer, or tablet.
- Literature reviews based on already included studies.

### Charting of the data

Following the recommendations of Arksey and O’Malley [14], data from the included articles were extracted and organised into tables according to the following predetermined categories: author(s), year of publication, country; study objective; study design; population; basis for guideline (if applicable); examination components; main findings.

### Summary and reporting of results

To provide an overview and allow for analysis of potential patterns and in adherence to Arksey and O’Malley’s [14] recommendation for consistent reporting of findings, results were compiled in tabular form. Since the included articles varied in nature, we created two separate tables—one for articles presenting recommendations or guidelines, and one for articles describing validity and reliability studies. The study objectives are reported in full, while the results are presented only insofar as they relate to the aim of this scoping review. Additionally, the results are presented and synthesised under the following headings: *Preparations for Video-Based Examination*,* Medical history*,* Inspection*,* Palpation*,* Range of Motion Assessment*,* Muscle Strength Assessment*,* Neurological Assessment*,* Specific Shoulder Tests*,* Diagnosis*,* and Pediatric Examination.*

## Results

**Fig. 1:**
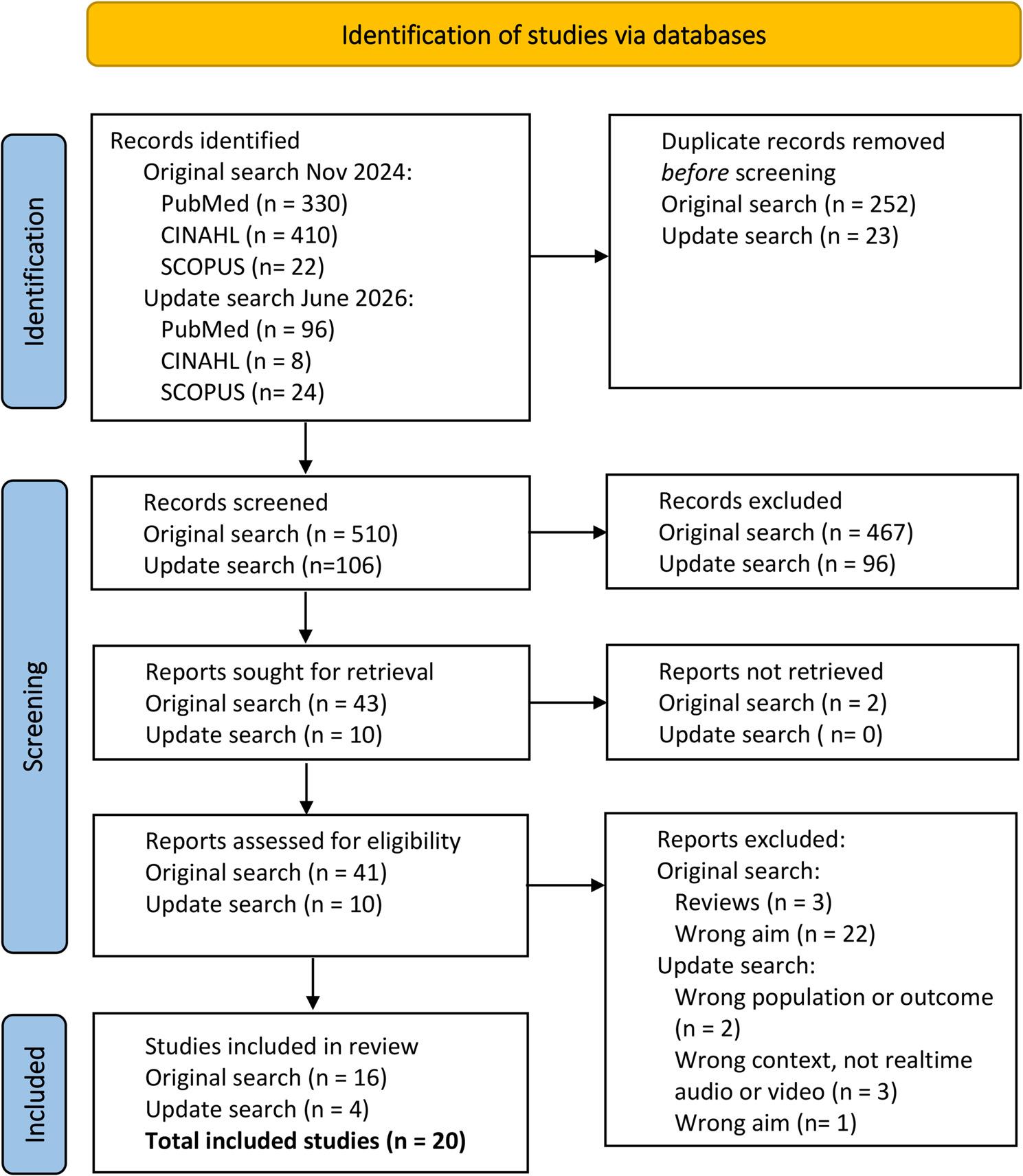
Description of the selection process and its results. From: Page MJ, McKenzie JE, Bossuyt PM, et al. The PRISMA 2020 statement: an updated guideline for457reporting systematic reviews. BMJ 2021;372:n71. DOI: 10.1136/bmj.n71

The searches in the three databases yielded a total of 762 hits, which were reduced to 510 after the removal of duplicates. After screening of titles and abstracts, 41 articles remained for full-text review. Of these, 16 articles met the inclusion and exclusion criteria and were ultimately included in this scoping review. The updated search yielded 129 records, reduced to 106 records after duplicate removal. After screening of titles and abstracts, 10 articles were retained for full-text review, of which four were included (Fig. 1).

### Included studies

A total of 20 articles were included in this scoping review. Most articles were published from 2020 onwards [19–30], with only four articles published before 2020 [13, 31–33]. The oldest article was published in 2007 [31]. Ten articles originated from North America [19–21, 24, 25, 27, 30, 34, 35], while the remaining articles came from Australia [13, 31, 32], Israel [26, 33], India [29], Brazil [27, 36, 37], and Italy [22]. Seven articles were designed as recommendations and guidelines (Table 1), twelve were validity and/or reliability assessments of different examination components, specific tests, or measurements within a video-based shoulder examination (Table 2), and one was a case report [25]. The guideline articles aimed to support clinicians in conducting video-based examinations and described procedures for examination, often including images and guidance regarding the structure and various elements of the examination [19–23, 28, 30]. One of the guideline articles described an examination protocol evaluated in a clinical setting with respect to the examiner’s perceived ability to perform specific shoulder tests in a video-based examination [23]. The validity/reliability studies [13, 24, 26, 27, 29, 31–37] investigated examination components, specific tests, or measurements within a video-based shoulder examination (Table 2). Two of the validity/reliability articles exclusively investigated internet-based/app-based goniometric measurement [29, 31]. Eight articles examined the agreement in diagnosing and assessing shoulder problems between video-based and conventional face-to-face examinations [13, 24, 26, 32, 34–37]. The case report described the examination of a single patient [25].

**Table 1:** Overview of included guideline articles

Author, year, country	Objective	Basis for guideline	Description of video-based shoulder examination
Applewhite et al., 2022, USA [19]	Serve as reference material for clinicians new to digital shoulder assessments.	Not specified.	• Preparation • History taking • Inspection • Palpation • ROM assessment (active mobility and goniometric measurement) • Muscle strength assessment • Specific shoulder tests
Boisvert-Plante et al., 2021, Canada [20]	Provide written guidance for clinicians on conducting telemedicine-based assessments of the neck and upper limbs in children aged 3–18.	Based on a literature review examining clinical outcome comparability between digital and face-to-face assessment. Includes 11 shoulder articles and some validity/reliability discussion.	• Preparation • Cervical spine assessment • Inspection • Self-palpation • Shoulder ROM (active + goniometric) • Sensory assessment • Muscle strength assessment • Specific shoulder tests • Patient-reported outcome measures for children
Lamplot et al., 2020, USA [18]	Provide a guide for digital examination, including verbal instructions for patients, suggested multimedia tools, and documentation checklist.	Not specified. Authors acknowledge that test modifications likely affect sensitivity and specificity.	• Preparation • Inspection of painful area • ROM assessment (active and functional movements) • Sensory assessment • Muscle strength assessment • Specific shoulder tests
Laskowski et al., 2020, USA [17]	Provide clinicians with guidelines for telemedicine-based musculoskeletal examinations of the shoulder, knee, hip, ankle, cervical and lumbar spine.	Based on validated face-to-face test methods, modified for patient self-performance. Authors note lack of data on sensitivity and specificity for digital adaptation.	• Preparation • Inspection: posture, functional movement • Self-palpation • Active ROM assessment • Muscle strength assessment • Specific shoulder tests
Moretti et al., 2023, Italy [21]	Develop a model for remote shoulder examination using traditional tests and new tech tools.	Observational study: 10 orthopaedic surgeons examined 6 patients both in-person and via webcam (WhatsApp).	• Preparation • Inspection • Self-palpation • Active ROM assessment • Specific tests with comparison to in-clinic performance
Phuphanic et al., 2021, USA [28]	Offer practical guidance for telehealth consultations focused on physical examination.	Based on previously published video assessment guidelines, combined and adapted.	• Preparation • Inspection • Active ROM assessment • Self-palpation • Specific shoulder tests
Wahezi et al., 2020, USA [26]	Describe and illustrate MSK and neuro exam techniques adapted for telemedicine. Provide guidance for pain and functional assessment in spine, hips, shoulders, and knees.	Consensus-based guidelines from 20 multidisciplinary experts (PM&R, ortho, neuro, rheum, anaesthesia).	• Preparation • History taking • Inspection • Self-palpation • Specific shoulder tests • ROM assessment (active + goniometric) • Muscle strength assessment

**Table 2:** Overview of included validity and reliability studies

Author, Year, Country	Objective	Study population	Study design	Description of video-based examination	Main findings
Beraldo et al., 2025, Brazil (Diagn. Acc.) [36]	Evaluate the diagnostic performance of virtual (telemedicine) shoulder maneuvers against MRI, with secondary analyses including agreement between virtual and in-person examination.	32 patients with rotator cuff syndrome (mean age 59.6 y). This sample represents the telemedicine arm of the study below.	Cross-sectional diagnostic accuracy study. Order of examination randomized.	• Specific shoulder tests (modifications described)	Against MRI, telemedicine tests were highly sensitive: Jobe pain sensitivity 96.2%, infraspinatus weakness sensitivity 76.9%, Bear Hug weakness sensitivity 57.1%. In-person tests showed similar sensitivity for most maneuvers, with a tendency to classify fewer tendons as weak in MRI-negative shoulders. Telemedicine more sensitive for muscle weakness. Agreement between modalities was excellent for pain (κ up to 1.00) and moderate for weakness (κ 0.11–0.47).
Beraldo et al., 2025, Brazil (Reliability) [37]	Evaluate interrater reliability and reproducibility of treatment indications using telemedicine, assess reproducibility and accuracy of physical examination tests, and examine level of patient satisfaction.	64 patients with rotator cuff syndrome (32 patients in each group). Mean age 59.6 y (telemedicine group) and 60.3 (in-person group)	Cross-sectional diagnostic accuracy study. Participants underwent two appointments conducted by different orthopaedic surgeons. Order of consultation randomised.	• Specific shoulder tests for pain and loss of strength	Reproducibility of tele-consultation for treatment indication high in both groups; κ 0.82 to 0.93. For physical examination tests, reproducibility was high in the in-person group (κ 0.63 to 1), but varied in the telemedicine group (κ 0.11 to 1). Test accuracy in the in-person group was high (sensitivity 0.94 to 1; specificity 0.66 to 1), whereas the telemedicine group showed greater variability (sensitivity 0.60 to 1; specificity 0.16 to 1). Patient satisfaction high in both groups, no significant difference (*p* > .676).
Bradley et al., 2021, USA [24]	Compare diagnostic accuracy between simulated digital vs. in-clinic assessment for rotator cuff tears; assess validity of specific shoulder tests.	60 individuals (age > 40) with shoulder pain. 50 underwent MRI for confirmation.	Case-control study. Patients examined by orthopaedists both in clinic and digitally. Each orthopaedist examined a patient once.	• Active ROM assessment • Muscle strength assessment • Specific shoulder tests (modifications described)	Diagnostic agreement with MRI: high (clinic 45.53%, digital 45.72%). Moderate agreement on painful arc, shrugging, and internal rotation (κ 0.42–0.59). Statistically significant.
Cottrell et al., 2018, Australia [32]	Assess diagnostic agreement between clinical and video-based examination for MSK disorders in lower back, shoulder, and knee. Parameters: diagnosis, treatment/management, need for further investigation.	42 individuals with chronic non-acute MSK issues, 14 with shoulder disorders.	Randomised order. All patients underwent both in-person and video examinations. Six physiotherapists, no one examined same patient twice.	• Individually tailored examination (procedure not described).	Diagnosis agreement for shoulder: same 28.6%, similar 50%, different 21.4%. Treatment/management agreement: 71.4%. Imaging need agreement: 92.9%.
Glover et al., 2025, USA, [34]	Compare diagnostic accuracy of telehealth diagnostic examinations for pathologies of the shoulder against an in-person examination. (Expanded study of a data set included in the publication by Bradley et al. 2021)	62 patients (mean age 57.9 years ± 11.2). 60 patients obtaining an MRI	Case-control study. Patients underwent in-person standardized clinical examination (SCE) and standardized telehealth examin- ation (STE) during the same visit by two different providers in randomised order.	• Active and passive ROM assessment • Muscle strength assessment • Specific shoulder tests for pain and loss of strength (modifications described)	No significant differences in the pooled diagnostic accuracy of identifying rotator cuff tears, glenohumeral arthritis, or AC arthropathy between SCE and STE (*p*=.495, 0.469, 0.333, respectively). Moderate to substantial agreement for selective tests. Highest agreement between SCE and STE for the shoulder shrug test, night pain, and internal rotation limitation for identifying RCT.
Goldstein et al., 2019, Israel [33]	Evaluate the validity of the Constant Score for video-based shoulder examinations.	51 participants (47 completed), mean age 43 (range 19–80).	Cross-sectional/prospective. All had both in-person and Android-based digital examinations by different orthopaedists.	• Constant Score (pain, activity level, arm position, active ROM, muscle strength)	Very high agreement (mean score 71 clinic vs. 72 video). 95% of video scores within 7 points of clinical scores. Highest agreement at score extremes.
Gushikem et al., 2022, Brazil [27]	Assess validity of video-based strength, ROM, sensation, and Tinel sign assessment in traumatic brachial plexus injury.	21 participants (mean age 29.3) with TBPI.	Cross-sectional. One physiotherapist and one neurosurgeon conducted both types of assessments. No repeated examiners.	• Active and passive ROM in shoulder, elbow, wrist • Muscle strength • Sensation (camera angles adjusted, relative assisted)	High strength agreement for trapezius and abductors (ICC 0.79, 0.87). ROM moderate-high agreement (70–80%, κ 0.29–0.55). High sensory agreement C6–C8 (κ 0.74–0.83), low for C5 (κ 0.19). Tinel sign moderate (kappa 0.57).
Hoffman et al., 2007, Australia [31]	Assess validity and reliability of internet-based goniometer for measuring upper limb ROM in post-stroke patients.	10 hospitalised stroke patients (mean age 70, 80% male).	Controlled hospital setting. Traditional and digital goniometry, with video recording for reliability analysis.	• Active ROM in shoulder flexion and abduction using universal and digital goniometer • Camera placement described	High agreement between methods (*r* > .99). Mean difference ≤ 2.1°. High intra/inter-rater reliability (ICC 0.97–0.99).
Rabin et al., 2020, Israel [26]	Assess agreement in diagnosis, treatment decisions, and further testing needs between video and clinical shoulder examinations. Also describe exam features and patient satisfaction.	47 individuals (mean age 44.6 ± 22) with varied shoulder complaints.	Patients examined by two orthopaedists both in person and via smartphone (three missed digital examination). No repeated examiner-patient pairs.	• Assistant preparation (camera handling) • Functional movement • Active and passive ROM • Muscle strength • Modified specific tests	Diagnosis agreement: 85.1% (κ 0.82). Treatment decision agreement: 61.7% (κ 0.43). Testing need agreement: 74.5% (κ 0.49). Digital examination showed less passive ROM and took longer.
Sahu et al., 2021, India [29]	Evaluate precision and reliability of app-based goniometry compared with traditional goniometer for shoulder ROM.	24 individuals (22–43 y/o) without shoulder pain and 16 patients (24–68 y/o) with shoulder complaints.	Validity/reliability study. Same-day in-clinic and Zoom examinations. Videos sent to two raters using app.	• Active ROM (elevation, abduction, internal/external rotation) • Camera positioning and movement described	High agreement between tools (ICC 0.74–0.98). App-based method had high reliability (ICC 0.86–0.99). Slightly lower inter-rater reliability for traditional tool (ICC 0.70–0.96). Mean difference: 0.2°–7°.
Sheth et al., 2025, Canada [35]	Examine the intra- and inter-examiner agreement of clinical tests and a management plan between a virtual video encounter and an in-person assessment in patients seen at a specialty shoulder program at an academic center.	30 patients with shoulder pain (rotator cuff impingement or tear, biceps pathology, arthritis, and adhesive capsulitis), 14 (47%) females, mean age: 57.5 (SD 11) y,	Repeated measures design, virtual assessment first.	• Cervical spine movements • Shoulder ROM • Clinical tests • Operative and non-operative management	ICC 0.83 to 0.87 for shoulder ROM, indicating good reliability. Cervical spine Spurling test had an almost perfect agreement (κ 0.83). κ values were substantial for the hornblower sign (κ 0.78), painful arc (κ 0.65), lift-off test (κ 0.65), and belly press test (κ 0.61). Agreement was moderate for the cervical spine ROM (κ 0.60), Jobe’s test (κ 0.57), Hawkins-Kennedy (κ 0.52), and cross-body adduction (κ 0.47). The management plan showed almost perfect agreement with respect to conservative vs. surgical management (κ 0.83). Percentage of inter-examiner agreement 0.80 to 1.00.
Steele et al., 2012, Australia [13]	Evaluate the use of a telerehabilitation system to formulate valid and reliable diagnoses for shoulder disorders; assess validity and reliability of specific examination findings; examine patient satisfaction.	22 individuals with shoulder problems (28 assessments due to bilateral issues). Mean age: 30.7 ± 14.2.	All participants were examined by three physiotherapy students in-clinic and via video, in randomised order. Recordings were used for validity and reliability assessments by experienced clinicians.	• Inspection • Active ROM assessment • Muscle strength assessment • Self-palpation • Specific shoulder tests	Diagnostic agreement: moderate-high (59.72% and 78.6%). Diagnostic reliability: 73.08% interrater, 100% intrarater. High agreement for examination findings, best for ROM (87.4%), lowest for nerve testing (56.1%). Statistically significant.

*ICC* Intraclass correlation coefficient, *κ* kappa, *ROM* Range of motion

### Description of the included case report

Wang et al. (25) described the management of a rotator cuff injury in a teenage semi-professional tennis player. The article outlined the examination, assessment, and rehabilitation carried out virtually. The video-based examination consisted of active ROM measurement using screenshots, testing of muscle strength with resistance, and specific shoulder tests.

### Preparations for the video-based examination

Several articles described in detail how both healthcare providers and patients can prepare for a video-based examination of the shoulder to ensure the highest possible quality [19–23, 26, 28, 30]. The camera constituted a central component of the examination. Four articles [21, 23, 27, 28] recommended a camera distance of between 1.5 and 1.8 m from the patient. Lamplot et al. [20] differed from the other articles in suggesting that the camera should be placed 3–4.5 m away from the patient. Other articles [19, 20, 22, 26, 30, 35] provided more general guidelines for camera positioning, such as ensuring that the relevant body part is fully visible, that the entire body is seen in the frame, or that the camera is at eye level if the patient is seated. Some of the articles reported that instructions were provided prior to examinations via video without providing further detail [34, 36, 37].

Patients are expected to wear appropriate clothing, such as a t-shirt or tank top, or no shirt at all, to facilitate the examination as much as possible [19–23, 28, 30, 35]. Four of the articles recommended that patients receive detailed pre-visit instructions from the healthcare provider in order to minimise the time spent on non-clinical guidance during the consultation and to prepare the patient for how the examination will be conducted [20, 24, 25, 28].

### Medical history taking

One article [13] emphasised that medical history taking conducted virtually largely follow the same structure as in a traditional consultation. Two of the included articles [19, 20] highlighted the importance of the patient providing detailed information prior to a virtual consultation. This information should include the patient’s primary complaint, medical history, surgical history, allergies, current medications, social factors, and a systematic review of body systems. The remaining articles addressed history taking only briefly or not at all.

### Inspection

Several articles discussed the inspection of the shoulder in terms of swelling/edema, asymmetries, redness, scapular winging, muscle atrophy, posture, or other visible abnormalities [19–23, 26, 28, 30]. The inspection involved a careful visual assessment of the shoulders from the front, back, and side perspectives. One of the studies also described how patients can conduct self-inspection of the shoulders using a mirror [21].

### Palpation

Patient self-palpation was mentioned in eight of the included articles [19–23, 26, 28, 30]. The extent of the descriptions varied greatly, and in two of the articles, palpation was described solely as the patient pointing to the area of pain in the shoulder [20, 28].

Two of the articles [19, 21] described self-palpation of the clavicle, the acromioclavicular joint out to the acromion, and the lateral aspect of the shoulder. The latter article included both an image and instructions for verbally guiding the patient. One article [23] recommended asking the patient about their perception of swelling, muscle contractures, deviations from normal anatomy, or specific pain in areas such as the long head of the biceps tendon or the acromioclavicular joint.

The most detailed descriptions were provided in the articles by Boisvert-Plante et al. [22] and Phuphanic et al. [30]. They described that self-palpation can be performed over the coracoid process, the long head of the biceps tendon, posteriorly over the scapular spine, or over the trapezius, in addition to the areas described in other articles. Boisvert-Plante et al. [22] also noted that palpation can be performed by a guardian or assisting adult, but emphasised that anatomical knowledge, palpation skill, and the ability to gauge pressure are required, as otherwise palpation may be perceived as unsafe or overly forceful.

### Assessment of range of motion

Examination and assessment of active ROM in the shoulder joint were described in most of the articles [19–31, 33], although the methods vary. Five articles [26, 27, 34, 35, 38] addressed the assessment of passive ROM. One of these articles [26], described how the patient uses their healthy arm to raise the arm being assessed, and one described using a stick [34]. Visual assessments were amongst the most commonly used methods in clinical evaluations, as noted in four of the included articles [19, 22, 27, 33]. One of the included articles [27] reported that visual ROM assessment of shoulder abduction via video had an 80% agreement with conventional in-clinic examination, while shoulder flexion and external rotation did not show significant agreement due to methodological and technical factors. A pooled accuracy of 70.8–84.6% was presented for passive external rotation and forward flexion [34]. Another article [13] reported an agreement of 87.4% for visual estimation of ROM between video-based and face-to-face examinations, and ICC between 0.83 and 0.87 was found for active flexion, abduction, and external rotation [35].

In addition to visual observation and assessment, there were digital applications and web-based goniometers used for objective ROM measurement. This was described to varying degrees in five of the included articles [21, 22, 25, 29, 31]. Two of these [29, 31] investigated the validity and reliability of video-based measuring tools, with the results showing minor deviations compared to manual goniometers and mean differences ranging from 0.2°–7° and 1.1°–2.4°, respectively.

The patient’s position during ROM assessment varied depending on the specific movement being evaluated and includes standing, sitting, and lying positions, regardless of whether the assessment concerns active or passive ROM [19–21, 24, 26–31]. None of the included articles investigated which position is most useful or valid for ROM assessment.

### Assessment of muscle strength

The majority of the included articles described one or more methods for assessing muscle strength in video-based examinations. One method described in several articles involved the patient applying resistance using their healthy arm [19, 22, 24, 26, 27, 30, 34, 36]. Another commonly used method involved the use of external objects such as household items, doorframes, or tables to create resistance [19–24, 26, 28]. An additional method described is manual muscle testing (MMT), where an assisting person applies resistance to evaluate muscle strength [19, 22, 24]. In such cases, the examining clinician gives detailed instructions to the assisting individual, especially regarding how the applied pressure should be delivered [25].

Two of the included articles [22, 27] used a standardised 0 to 5 scale to assess muscle strength. One article [22] described that patients can use weights between 5 and 10 pounds (approximately 2–4 kg) to indicate a strength level of at least 4 out of 5, and to potentially reveal asymmetries in muscle strength. The same article suggested a modified strength scale, in which patients were assessed as having 5 out of 5 strength if they could perform shoulder abduction with a weight exceeding 10 pounds (approximately 4 kg). Another article (27) described the difference between strength grades 4 and 5 based on observational analysis, where grade 5 is characterised by the inability to depress the tested body part during the evaluation. Three studies used specific tests to identify weakness [35–37]. In the remaining studies, the assessment was based on the patient’s own perception, as the clinician asks whether the patient experiences pain or unusual weakness during muscle activation [19, 23–25, 28, 34].

### Neurological examination

Neurological examination was addressed in seven of the included articles. Four articles [13, 20–22] recommended performing a cervical spine examination in conjunction with a shoulder assessment. Applewhite et al. [21] advised that if symptoms may originate from the neck, the patient should schedule a new appointment for a face-to-face examination. More in-depth testing of sensory and motor function, by assessing muscle strength in each innervation area, was described by Boisvert-Plante (22). This article also discussed how a third party may assist in the testing. In the article by Gushikem [27], the validity of sensory testing was evaluated, showing good agreement between video-based and face-to-face examinations for innervation levels C6, C7, and C8 (kappa 0.74–0.83), but poorer agreement for C5 (kappa 0.19). Four articles described specific nerve tests: the Roos test for Thoracic Outlet Syndrome [20, 22, 30] and the Tinel’s test [27]. In these tests, the patient is guided verbally and visually; for Tinel’s test, the patient performs percussion on the nerve themselves. Steele et al. [13] suggested including nerve testing during the shoulder examination but did not describe how it should be conducted. In the discussion section, the authors noted that the validity of nerve testing was lower compared to other tests, which may be due to the complexity of nerve dynamic tests that involve multiple intricate movements that are difficult to explain and demonstrate in a video-based examination.

### Specific shoulder tests

A total of 15 of the included articles addressed specific shoulder or orthopaedic tests [13, 19–26, 28, 30, 34–37] (Additional file 3). Some articles described a full test battery, while others presented individual tests. Several articles [22, 24, 28, 34] indicated for each test whether any modifications are needed or if assistance is required to perform the test virtually. Other articles did not mention any modifications. When modifications were described, they consistently involved the patient applying resistance themselves instead of the examiner doing so. Resistance may be applied with the opposite hand, such as in Speed’s test [19], or by using a table, door frame, or some form of weight [20].

Six articles [13, 24, 34–37] evaluated the agreement, reliability, or diagnostic accuracy of multiple specific shoulder tests performed during video-based assessment. In one of these studies [13], diagnostic performance was reported only for the overall examination, without presenting results for individual clinical tests. Overall agreement between video-based and face-to-face examinations was moderate [13, 24]. Three studies reported agreement for individual shoulder tests and consistently found higher agreement for pain-provocation and movement-based tests than for strength-based tests, with agreement ranging from moderate to almost perfect depending on the specific test [34, 35, 37].

The article by Bradley et al. [24] showed that tests requiring less involvement from an examiner had higher agreement, while tests that required explanation or subjective grading showed lower agreement. The tests demonstrated poor to moderate agreement (Kappa 0.07–0.87) between video-based and face-to-face examinations. In the article by Moretti et al. [23], ten examiners assessed the feasibility of performing shoulder tests virtually. The general opinion was that stability tests could not be performed in a video-based examination; only the Apprehension test was deemed feasible by most examiners.

In four of the included articles [23–25, 28], movements such as painful arc, hand-behind-neck, and hand-behind-back were described as tests or examination maneuvers. However, as these movements are partly or entirely included in the inspection and measurement of active ROM, they were not considered specific tests in this review.

### Diagnosis

Four of the included articles addressed the level of agreement in diagnoses made via video-based examination compared to conventional face-to-face examination. One article [26] specifically examined agreement in diagnosing rotator cuff rupture and reported no significant difference in diagnostic accuracy; both video-based and face-to-face examinations often resulted in incorrect diagnoses. In two other articles, the same or similar diagnoses were made in 78.6% [32] and 85.1% [26] of cases, respectively.

Steele et al. [13] showed moderate agreement (58.7%) when diagnoses were made regarding specific anatomical structures (e.g., supraspinatus rupture) or specific conditions (e.g., adhesive capsulitis), and substantial agreement (78.6%) when diagnoses were categorised more broadly by anatomical system (e.g., skeletal, muscular, neural, joint, or other origin of symptoms). Two additional studies compared video-based examination with MRI as the reference standard and reported diagnostic accuracy comparable to face-to-face assessment overall [34, 36]. However, diagnostic performance varied across individual clinical tests, with pain-provocation and movement-based tests generally demonstrating higher diagnostic accuracy than strength-based tests, which showed greater variability in sensitivity, specificity, and overall diagnostic performance [34, 36].

### Pediatric examination

One of the included articles [22] focused on pediatric examination of the neck and shoulder in children aged 3–18 years. Particular emphasis was placed on the role of the assisting caregiver during the examination, for instance, to apply resistance during a movement or to position the child in front of the camera. Teenagers were generally able to perform examination tasks similarly to adults, such as self-palpation or applying resistance themselves during strength testing.

When testing range of motion, one challenge described was getting younger children into the correct position, especially for measurement using an internet-based goniometer. A suggested solution was to estimate mobility by having the child reach for toys in different directions [19, 22].

## Discussion

The main findings of this scoping review show that there is a growing literature base describing real-time, video-based examination of the shoulder, and that there are examples of procedures covering most aspects of such an examination. The results highlight similarities in the description of examination procedures, with multiple articles describing the preparations and structure of the examination in a similar way. The majority of the included articles also examined assessment of active ROM and various methods for evaluating muscle strength. Our review also reveals differences, for example in the procedures used for muscle strength assessments, in modifications of shoulder tests, and in whether or not the examination includes evaluation of nerve function. Furthermore, our results indicate a moderate-to-high diagnostic agreement between video-based and in-clinic examinations. Our review also identifies knowledge gaps in the field and highlights the need for more research, particularly regarding validity.

Our findings are substantially consistent with the results from two previous reviews concerning video consultations for musculoskeletal rehabilitation of upper extremity conditions [11, 12]. Maltby et al. [12] found that ROM was the most common component assessed in video-based examinations, and that benefits with this mode of assessment included time and cost savings, maintaining patient–therapist relationships, and increasing patient independence. They also found that most diagnostic assessments, except joint and nerve tension tests, could be considered valid and reliable. Zischke et al. [11] found in their systematic review that video-based shoulder assessment was valid and reliable for specific assessment components, particularly ROM, special orthopaedic tests, pain response, muscle strength, and diagnosis and management decisions. They also found that participants generally reported high satisfaction with the video mode, although, if given a choice, they would prefer to see a clinician in person. The video assessment mode was also found to be relatively cost-effective, especially in rural settings. Hence, both our and previous reviews’ findings suggest that there is a viable place for video-based shoulder assessment as a complement to conventional, face-to-face assessment. Amongst the articles included in our review, most investigated the agreement and validity of specific examination procedures [13, 24, 27, 29, 31, 33–37]. However, not all articles addressed the same examination components, and some combined results from several different procedures or tests. In addition, the articles used different starting positions for the patient and different methods of applying resistance during muscle strength assessments. Based on ours and others’ findings, further research is needed to allow for comparison of study results and to draw conclusions regarding the best methods for conducting a video-based examination.

Several articles differ in their descriptions of the use of specific shoulder tests. This is particularly the case with stability tests of the shoulder. Moretti et al. [23], for example, state that only the apprehension test, amongst several possible stability tests, can be carried out in a virtual examination, and that clear signs of instability should be assessed in a clinic setting. Applewhite et al. [21] advise against conducting stability tests during video-based examinations altogether, although they do not provide a rationale for this. In contrast, Boisvert-Plante et al. [22], in their guidelines, include tests for instability in all directions and describe how a parent or assisting caregiver can carry out these tests during a video-based examination [22]. Previous research on shoulder dislocations has shown a high risk of recurrent instability, with the major risk factors being youth, male sex, and participation in contact sports [39]. Fedorka and Mulcahey [40] emphasise the importance of a thorough clinical examination to distinguish between general joint laxity and true instability, and note that comparison between the patient’s shoulders is critical to the assessment. The fact that there is a risk of recurrent instability, that the examination is important, and that there are significant differences in recommendations and guidance, argues for a cautious approach to performing shoulder stability tests during video-based examinations. More research is needed regarding the potential risks of performing these tests via video in order for clinicians to feel confident using them in virtual healthcare consultations.

In relation to ROM measurements using app-based or internet-based goniometers, the results of the two articles that examined this [29, 31] show high validity and reliability. This finding is consistent with those of a previous systematic review by Shepherd et al. [41] on digital goniometric measurement that included various technologies. However, another recent systematic review by Chen et al. [38] found that remote measurement methods of shoulder motion consistently showed an overestimation of ROM compared to reference standards, with self-measurements showing lower and non-significant bias compared to assessor-guided methods. Even so, the results suggest that digital methods for measuring ROM may be a useful alternative in musculoskeletal examinations. The most accessible way for assessing ROM in a video-based examination is by visual estimation. Our review shows that this also has high validity, and although further research may be needed to identify optimal performance, the findings indicate that visual estimation can be used in video-based examinations.

When discussing the need for validity studies concerning virtual examinations and their individual components, it is important to bear in mind that there are also validity challenges in traditional shoulder examinations conducted in clinic. This has previously been demonstrated by, amongst others, de Winter et al. [42], who report poorer inter-rater agreement amongst physiotherapists assessing shoulder conditions in patients with severe pain, multiple issues, or bilateral problems [42]. As the pathology and structural causes of certain conditions are not always clearly understood, examiners may sometimes arrive at different diagnoses—or even incorrect ones—even when a full physical examination is carried out in a clinical setting [13]. The findings of the study by Bradley et al. [24] show near-perfect agreement in the diagnosis of rotator cuff tears between video-based and traditional clinical examination. However, the results also show that examiners were incorrect in approximately half of the cases when their assessments were compared with imaging results. This could be seen as a critique of musculoskeletal and orthopaedic examination methods in clinics, but also as an opportunity to use alternatives such as video-based examination, as it leads to diagnostic accuracy comparable to that of physical examination. The latter conclusion is in line with research on the rehabilitation of musculoskeletal disorders via real-time virtual solutions, where the results of a systematic review and meta-analysis showed comparable outcomes between virtual rehabilitation and standard in-clinic rehabilitation for all measured outcomes [32]. The findings of this review on diagnosis and assessment of shoulder problems suggest that video-based examination could at least be used as a form of screening or to broadly define the patient’s complaints. However, the clinician must then determine whether further in-person examination is required to gather additional information necessary for making an informed assessment and treatment plan.

The primary strengths of this scoping review lie in its use of the PCC framework to formulate the research question and its adherence to recommended guidelines for the conduct and reporting of scoping reviews [14, 16]. A comprehensive search was conducted in three major databases, and the exclusion of grey literature was a deliberate choice to ensure quality [43]. The original search was updated shortly before the publication of this article, which led to the inclusion of four additional studies. Reference lists from excluded reviews were examined to identify additional relevant articles, contributing to the review’s comprehensiveness. However, limitations include the omission of the keyword “orthopaedics,” which led to the late discovery of a relevant article [43, 44], and the restriction to English-language publications, which may have narrowed the cultural and linguistic scope of the results. Another limitation is the absence of a quality assessment of the included studies. While common in scoping reviews, this omission may influence both the interpretation and applicability of the findings, as noted by Levac et al. [15] and Brien et al. [45]. Additionally, the failure to screen reference lists from all included studies may have resulted in missed articles. Nonetheless, our review successfully maps the current state of research, highlights significant knowledge gaps, and provides a foundation for future investigations.

### Implications for practice and research

Video-based methods for examining the shoulder joint may have the potential to serve as a complement to traditional clinical procedures when examining, assessing, and diagnosing musculoskeletal pain in the upper extremity. The articles included in this review demonstrate procedures for conducting video-based examinations and provide evaluations of the level of agreement between video-based and conventional clinical assessments that may serve as useful guidance. However, evidence remains insufficient regarding how the various components of the examination can best be conducted with quality and efficiency, something that practising clinicians need to be aware of and that merits a rigorously conducted systematic review. Amongst the various components, ROM assessment is the one currently supported by the strongest evidence.

To ensure high-quality care and comparability between different healthcare providers and clinical environments, the development of standardised protocols for video-based examinations is needed. Such protocols could help establish consistent practice and strengthen the usability and credibility of virtual care in the management of musculoskeletal disorders. The findings of this scoping review may serve as a point of departure for this process.

The field of video-based examination methods is still evolving. Further studies are required to establish reliability and clinical utility of conducting examination in this form, and this review may serve as a basis for future research. More studies are needed regarding the validity and reliability of the various components of a shoulder examination, particularly for the many specific shoulder tests currently available, with focus on the tests that have shown the highest sensitivity and specificity in their original, in-person, assessment mode. Moreover, further studies are warranted to explore the level of agreement in assessment and diagnosis between video-based and in-person, face-to-face shoulder examinations.

## Conclusions

This scoping review identified twenty studies outlining examination components and practical procedures for conducting video-based assessments of the shoulder. Our findings suggest that video-based shoulder assessment can play an important role as a complement to in-person, face-to-face, assessment of shoulder problems. Although the research field is growing, with multiple studies published in recent years, this review highlights remaining knowledge gaps, particularly on the validity of individual shoulder tests, accuracy of digital tools for measuring mobility, and consistency of diagnoses made via video-based versus in-person examinations. Further research on validation of virtual shoulder clinical examination is needed to determine best practices and the appropriate role of video-based assessments of musculoskeletal disorders of the upper extremity.

## Supplementary Information

Supplementary Material 1.

Supplementary Material 2.

Supplementary Material 3.

## Data Availability

All data relevant to the study are included in the article.
