# Supplementary material for: Video-based examination of patients with shoulder pain: a scoping review
Source: BMC Musculoskelet Disord. 2026 Jul 20;27:632. doi: 10.1186/s12891-026-10243-y (PMC13397946; doi:10.1186/s12891-026-10243-y)
Supplement: Supplementary file 2 — Supplementary Material 2. [file 12891_2026_10243_MOESM2_ESM.docx]

# **Appendix 2.**

## Search strategy in PubMed, search results in Pubmed, CINAHL and Scopus (*data for updated search in italics*).

| **Database** |  | **Search string** | **Results** | **Limits** | **Date** |
| --- | --- | --- | --- | --- | --- |
| **PubMed** | #1 | (Telehealth OR Telemedicine OR "Remote physical therapy" OR "Virtual physical therapy" OR Teleconsultation OR "Remote consultation" OR "Web-based" OR "Video examination" OR "VideoBased" OR "Video consultation" OR Videoconferencing) |  | None  *11 nov 2024-30 June 2026* | 11 november 2024  *Updated 30 June 2026* |
|  | #2 | (diagnos* OR exam* OR test* OR measur* OR evaluate*) |  | None  *11 nov 2024 - 30 June 2026* | 11 november 2024  *Updated 30 June 2026* |
|  | #3 | (Shoulder* OR "shoulder girdle") |  | None  *11 nov 2024 - 30 June 2026* | 11 november 2024  *Updated 30 June 2026* |
|  | #4 | #1 AND #2 AND #3 | 330  *96* | None  *11 nov 2024 - 30 June 2026* | 11 november 2024  *Updated 30 June 2026* |

**CINAHL**

Search date: 11 november 2024, *updated 30 June 2026*

Results: 410 records; *8 records in new search*

**Scopus**

Search date: 11 november 2024, *updated 30 June 2026*

Results: 22 records; *24 records in new search*
