# Supplementary material for: Video-based examination of patients with shoulder pain: a scoping review
Source: BMC Musculoskelet Disord. 2026 Jul 20;27:632. doi: 10.1186/s12891-026-10243-y (PMC13397946; doi:10.1186/s12891-026-10243-y)
Supplement: Supplementary file 3 — Supplementary Material 3. [file 12891_2026_10243_MOESM3_ESM.docx]

# **Appendix 3.**

## Specific shoulder tests described in included studies

| **Test** | **Examined structure** | **Mentions in article** |
| --- | --- | --- |
| Neer’s test | Impingement of the rotator cuff’s tendons | n=7 (17–20,22,26,34) |
| Hawkins-Kennedy test | Inklämning av rotatorcuffens senor vid akromion | n=12 (17–20,22–24,26,28,29,34,35) |
| Empty can/Jobe test | Supraspinatus | n=9 (18,19,21,24,26,28,36,37,35) |
| Resisted external rotation (liggande) | Infraspinatus and teres minor | n=7 (17,18,20,23,36,37,34) |
| Patte/Hornblower | Teres minor | n=3 (18,20,35) |
| Drop-arm | Rotatorcuff rupture | n=4 (20,22,23,34) |
| ER-lag-sign | Rotatorcuff rupture (supraspinatus) | n=3 (22,37,34) |
| Belly-press | Subscapularis | n=5 (19–21,28,35) |
| Lift-off/Gerber | Subscapularis | n=3 (20,34,35) |
| Scarf/Cross-body | AC joint pain | n=7 (17–20,26,28,35) |
| AC traction | AC joint pain and/or visible separeation of the joint | n=1 (28) |
| Shrug sign | Rörlighetstest, kan visa på artros och adhesiv kapsulit | n=2 (22,34) |
| Apprehension | Instabiliy of glenohumeral joint | n=6 (17–21,26) |
| Posterior stress test/Norwood | Instability of posterior glenohumeral joint | n=1 (18) |
| Sulcus-sign | Instabiliy of inferior glenohumeral joint | n=2 (20,28) |
| Load-shift/Drawer | Instability of anterior/posterior glenohumeral joint | n=2 (20,21) |
| Fulcrum and Jerk | Instability of glenohumeral joint | n=1 (21) |
| O´Briens test | SLAP injury | n=5 (17–20,24) |
| Crank test | SLAP injury/instability. Tested in external rotation position | n=1 (21) |
| Speed´s test | Biceps tendon injury | n=10 (17–21,24,26,39,34,35) |
| Yergason test | Biceps tendon injury | n=5 (17–20,35) |
| Upper-cut-test | Biceps tendon injury | n=1 (28) |
| Roos test | Thoracic outlet syndrome | n=3 (18,20,28) |
| Bear Hug test | Subscapularis | n=2 (36,37) |
